# Synchronizing the Liver Clock: Time-Restricted Feeding Aligns Rhythmic Gene Expression in Key Metabolic Pathways

**DOI:** 10.3390/cells15020193

**Published:** 2026-01-20

**Authors:** Shiyan Liu, Feng Zhang, Yiming Wang, Kailin Zhuo, Yingying Zhao

**Affiliations:** Department of Physiology, School of Basic Medical Sciences, Health Science Center, Shenzhen University, Shenzhen 518060, China

**Keywords:** circadian rhythm, obesity, time restricted feeding, metabolic disorders, liver

## Abstract

Non-alcoholic fatty liver disease (NAFLD) is closely linked to metabolic syndrome and circadian rhythm disruption, yet the mechanisms by which lifestyle interventions restore circadian organization remain incompletely understood. In this study, we employed a stringent 3 h time-restricted feeding (TRF) regimen in a mouse model of high-fat diet (HFD)-induced metabolic dysfunction. TRF markedly improved metabolic outcomes, including lipid accumulation, glucose tolerance, and behavioral and physiological rhythms. Importantly, through transcriptomic profiling using RNA sequencing, we found that TRF induced circadian rhythmicity in previously arrhythmic hepatic genes. This approach revealed that TRF promotes transcriptional synchronization within key metabolic pathways. Genes involved in autophagy, fatty acid metabolism, and protein catabolism exhibited coherent peak expression at defined time windows, suggesting that TRF temporally restructures gene networks to enhance metabolic efficiency. This intra-pathway synchronization likely minimizes energy waste and enables cells to execute specialized functions in a temporally optimized manner. Together, our findings identify temporal reorganization of metabolic pathways as a mechanistic basis for the benefits of TRF, providing new insight into circadian-based strategies for managing metabolic disease.

## 1. Introduction

Non-alcoholic fatty liver disease (NAFLD) has emerged as the most common chronic liver condition worldwide, affecting approximately 25% of the global population [1]. It encompasses a spectrum of liver disorders ranging from simple hepatic steatosis to non-alcoholic steatohepatitis (NASH), fibrosis, cirrhosis, and even hepatocellular carcinoma [2]. Despite its growing prevalence, effective pharmacological treatments for NAFLD remain limited, and current clinical strategies primarily focus on lifestyle interventions such as dietary modification and physical activity [3]. Increasing evidence suggests that circadian disruption—resulting from irregular sleep patterns, shift work, or mistimed feeding—plays a pivotal role in the development and progression of NAFLD. This link is supported by both human epidemiological studies and animal models, which show that perturbations in circadian rhythms can lead to hepatic lipid accumulation, insulin resistance, and systemic inflammation [4].

The liver is a highly rhythmic organ whose metabolic functions—including lipid synthesis, glucose metabolism, and bile acid turnover—are tightly regulated by the circadian clock. Notably, about 40% of hepatic transcripts exhibit circadian oscillations, underscoring the significance of temporal regulation in liver physiology [5]. This intrinsic rhythmicity is coordinated by core clock genes such as Bmal1, Clock, Per, and Cry, which interact with metabolic regulators to ensure time-of-day-specific gene expression. Disruption of the hepatic circadian clock—through genetic deletion of clock genes or environmental factors such as high-fat diet feeding, shift work, or irregular feeding patterns—has been shown to impair lipid homeostasis [6]. Recent studies have highlighted that circadian misalignment affects the expression of key genes involved in lipid homeostasis [7], including those regulating triglyceride synthesis and fatty acid oxidation [6,8]. Recent studies in both rodents and humans have demonstrated that circadian disruption exacerbates hepatic steatosis, alters the rhythmic expression of lipid metabolic genes (e.g., Srebp1c, Fasn, Cpt1a), and impairs the diurnal oscillation of key metabolic pathways, including autophagy and mitochondrial function [9,10].

Time-restricted feeding (TRF), which confines food intake to specific windows during the day without reducing caloric intake, has emerged as a promising non-pharmacological intervention for improving metabolic health [11,12,13,14,15,16]. Classic caloric restriction (CR) studies have indicated that under approximately 30% caloric restriction conditions, mice will naturally compress their voluntary feeding into a 2–3 h window [17,18]. Although the metabolic benefits of 6–12 h TRF have been well-supported by the existing literature [11,12,15,16,19], this study aims to explore a scientific question that has not been fully investigated: whether a more compact and extreme feeding window would intensify the potential circadian rhythm reprogramming effects?

We hypothesize that a shorter and more potent time-syncing signal (zeitgeber) can not only restore the rhythms of individual genes but also maximize the temporal reorganization of hepatic metabolic pathways. Animal studies have demonstrated that TRF can prevent obesity, insulin resistance, and hepatic steatosis induced by high-fat diets, even without weight loss [20,21]. These effects are thought to be mediated, at least in part, through the restoration of circadian rhythmicity in peripheral metabolic tissues, including the liver [22]. Beyond metabolic benefits, TRF has also been shown to extend lifespan in model organisms. Notably, this longevity effect critically depends on the integrity of the circadian clock and requires proper alignment between feeding–fasting cycles and endogenous biological rhythms. Misalignment between TRF timing and circadian system abolishes the life-extending effect [23]. Interestingly, recent studies suggest that TRF can still confer metabolic protection even in the absence of a functional circadian clock, likely by modulating the metabolic states of multiple organs—including adipose tissue, liver, gut, and the immune system. This observation implies that feeding alone can act as a system time cues sufficient to coordinate inter-organ metabolic homeostasis [24]. Importantly, feeding itself has been shown to influence the hepatic circadian clock, which is not merely a passive recipient of systemic signals but an active coordinator of rhythmic metabolic outputs. The liver clock translates feeding-related signals into systemic time cues and transmits them via non-cell-autonomous mechanisms to other peripheral tissues such as adipose tissue, skeletal muscle, and kidney. This inter-tissue signaling modulates the rhythmic expression of metabolic genes and is critical for maintaining whole-body energy homeostasis [25]. These findings highlight inter-organ rhythmic synchrony as a key feature of organismal metabolic health [26,27]. This inter-organ rhythmic synchrony relies on a hierarchical system: the central pacemaker in the hypothalamic suprachiasmatic nucleus (SCN) receives photic signals and coordinates peripheral organ clocks in tissues such as liver, adipose tissue, and skeletal muscle [28,29]. At its core, this system requires phase coherence in the oscillations of clock genes (e.g., Bmal1, Clock, Per, Cry) across different tissues to drive the temporally ordered expression of metabolic genes [30]. For instance, when hepatic clocks become misaligned with the central SCN clock, it exacerbates diet-induced obesity and hepatic steatosis; conversely, genetic re-synchronization of these clocks can protect metabolic health even in the presence of disrupted external behavioral rhythms [28]. This evidence indicates that internal clock synchrony within the organism may be more critical for maintaining metabolic homeostasis than strict alignment with external environmental cues [28]. This synchrony is manifested not only at the transcriptional level but also through rhythmic fluctuations of metabolites that mediate inter-organ “cross-talk”. The central clock primarily drives rhythmic behaviors such as feeding, which orchestrates over half of the circulating metabolite rhythms [29]. Concurrently, peripheral organs (e.g., liver) contribute tissue-specific metabolic rhythms, while the hepatic clock itself coordinates the temporal expression of metabolic genes across its cellular constituents (e.g., hepatocytes) [29]. Metabolites including NAD^+^, bile acids, and gut microbiota-derived products further feedback to regulate peripheral tissue clock gene expression, forming a bidirectional communication network [31]. Consequently, circadian disruption fundamentally impairs this spatiotemporal regulation of gene expression and metabolic networks across organs and cell types, leading to dysregulated energy partitioning and pathogenesis of metabolic syndrome and non-alcoholic fatty liver disease [30]. While foundational studies have demonstrated that TRF can improve metabolic parameters and reprogram circadian gene expression in the liver [32,33], the specific role of TRF in metabolic pathways and the extent to which it reprograms hepatic gene expression remain to be further investigated.

In this study, we aimed to determine whether time-restricted feeding (TRF) can reverse hepatic steatosis and reprogram circadian transcriptional rhythms as a mechanism of metabolic improvement. Using a mouse model of diet-induced non-alcoholic fatty liver disease, we implemented a 3 h TRF intervention and assessed its effects on liver histology, metabolic parameters, behavioral rhythms, and hepatic gene expression over a 24 h cycle. Our findings revealed that TRF not only alleviates hepatic lipid accumulation and improves systemic glucose metabolism but also restores diurnal patterns in hormone secretion, activity, and body temperature. Importantly, transcriptomic analysis demonstrated that TRF not only increased the number of rhythmically expressed genes but also induced temporal synchronization of gene expression within key metabolic pathways. Genes involved in autophagy, fatty acid metabolism, and proteostasis exhibited coordinated peak expression within well-defined time windows, suggesting that TRF restructures the temporal organization of gene networks to enhance metabolic efficiency. This pathway-level synchronization likely reduces energetic waste and enables cells to perform specialized functions with optimized timing.

## 2. Materials and Methods

### 2.1. Mice

Eight- to ten-week-old male C57BL/6 mice were obtained from the Guangdong Medical Laboratory Animal Center and housed under specific-pathogen-free conditions. They were maintained on a 12 h light/12 h dark cycle (lights on at 7:00, ZT0), with free access to water and unrestricted movement. Sixty 8-week-old male C57BL/6 mice were randomly assigned to four groups: the control group (CON group), the control with Time-Restricted Feeding group (CON + TRF group), the high-fat diet group (HFD group), and the high-fat diet with time-restricted feeding group (HFD + TRF group) to minimize baseline bias. Mice in the CON and HFD groups had ad libitum access to the standard chow or high-fat diet, respectively, whereas CON + TRF and HFD + TRF mice were only provided access to their respective diets during the daily ZT12 (19:00) to ZT15 (22:00) window, with food removed at all other times. Following this feeding regimen for six months, daily food intake was recorded and body weight was measured every three days. After 22 weeks, mice at each time point were selected randomly from their respective groups and anesthetized with isoflurane at five distinct time points: ZT0 (07:00), ZT6 (13:00), ZT12 (19:00), ZT18 (01:00), and ZT24 (07:00); blood was then collected, followed by dissection during which liver tissues were harvested and either flash-frozen in liquid nitrogen, embedded in OCT (Optimal Cutting Temperature) compound for cryosectioning, or fixed in 10% neutral buffered formalin for preservation.

All animal experiments were conducted in accordance with protocols approved by the Institutional Animal Care and Use Committee of Shenzhen University Medical School (IACUC-202400026, 19 December 2023).

### 2.2. Food Composition

Mice were fed one of two diets:

Standard SPF Rodent Maintenance Diet: This diet (SPF-F02-001, SiPeiFu (Beijing) Biotechnology, Beijing, China) provided 3.01 kcal/g. The percentage of calories derived from macronutrients was as follows: fat (12.1%), protein (21.6%), and carbohydrates (64.6%);

High-Fat Diet (HFD): A 60% high-fat diet (D12492, Research Diets, New Brunswick, NJ, USA) was used, providing 5.24 kcal/g. This diet comprised 60% fat, 20% protein, and 20% carbohydrate-derived calories.

### 2.3. Body Temperature

After 8 weeks of feeding, three randomly selected mice from each group were randomly selected for rectal temperature measurements at 3 h intervals from ZT0 to ZT48. For each measurement time point, rectal temperature was measured three consecutive times using a mouse rectal thermometer (Jinuotai, Beijing, China). The mean of these three measurements for each mouse was calculated and used as the final value.

### 2.4. Locomotor Rhythm Recording

After 12 weeks of feeding, three randomly selected mice from each group were housed in behavioral monitoring cages for 7 days to record their activity–rest rhythms. Locomotor activity over the 7-day period was analyzed using Ethovision XT video tracking software (Nordes, Wageningen, The Netherlands). Spontaneous motor activity was defined as the distance traveled per unit of time (3 min).

### 2.5. Treadmill Fatigue Test

After 16 weeks of feeding, exercise capacity was assessed in three randomly selected mice from each group using a treadmill fatigue test. The treadmill fatigue test was performed according to an established protocol [34]. Briefly, mice underwent a 3-day progressive training regimen on a treadmill set at a 10° incline with a 0.5 mA electric shock grid at the base to encourage running. Following training, the formal test commenced at 12 m/min. The treadmill speed was increased by 1 m/min every 5 min. Fatigue was operationally defined as remaining in the “fatigue zone” (the treadmill belt section within approximately one body length of the shock grid) for 5 consecutive seconds. Upon meeting this criterion, the mouse was immediately removed, and its running time, distance, and final speed were recorded.

### 2.6. Intraperitoneal Glucose Tolerance Test (IPGTT)

After 20 weeks of feeding, we subjected mice in each group to a 16 h fasting period. At ZT12, mice received an intraperitoneal injection of a 20% glucose solution (2 g/kg body weight). Following disinfection, blood glucose levels from the tail vein were measured at 0, 15, 30, 60, 90, and 120 min post-injection using an ACCU-CHEK Performa glucometer.

### 2.7. Blood Tests

Mouse whole blood was clotted at room temperature for 1 h, then centrifuged at 5000 rpm for 15 min at 4 °C to obtain serum. The serum supernatant was diluted fivefold with physiological saline for biochemical analysis of total protein (TP), albumin (ALB), total cholesterol (TC), total triglycerides (TG), aspartate transaminase (AST), alanine aminotransferase (ALT), Glucose (Glu), High Density Lipoprotein Cholesterol (HDL-c), Low-density Lipoprotein Cholesterol (LDL-c) using a blood biochemistry analyzer (Mindray BS-220, Shenzhen, China).

### 2.8. Oil Red O Staining

Frozen sections were prepared from the liver OCT embedded samples of three randomly selected mice per group, with a section thickness of 10 μm. Frozen sections of mouse liver tissue were circled along the periphery using a histological marker pen and equilibrated at room temperature for 5 min in a humidified chamber. Sections were then rinsed in 60% isopropanol for 20 s, stained with Oil Red O working solution for 10 min, and subsequently rinsed again in 60% isopropanol for 30 s. Following a distilled water wash, sections were counterstained with hematoxylin for 1 min, rinsed again in distilled water, and differentiated by a quick dip (1–2 s) in 1% acid-alcohol. Sections were then washed under slow running tap water for 30 min and finally mounted in glycerin gelatin for microscopic examination. Each mouse randomly selected one liver staining section for microscopic imaging. Three random fields (images) were captured from each section under the microscope for analysis. Images were captured at a magnification of 200× (with a 20× objective) for representative overviews and quantitative analysis. The quantitative analysis of the slices was accomplished using the ImageJ (1.54p) software.

### 2.9. Enzyme-Linked Immunosorbent Assay (ELISA)

Serum levels of insulin (JL11459, Jianglai, China), leptin (JL11317, Jianglai, China), and melatonin (JL10087, Jianglai, China) were quantified using commercially available ELISA kits according to the manufacturer’s instructions. Mouse serum samples were diluted 5-fold prior to analysis. Leptin levels were determined using a sandwich ELISA format, while insulin and melatonin levels were measured using competitive ELISA formats.

### 2.10. RNA-Seq

At each of the five time points (ZT0, ZT6, ZT12, ZT18, and ZT24), liver tissues were harvested from three randomly selected mice in both the HFD and HFD + TRF groups for subsequent RNA sequencing. Liver RNA extraction, library preparation (using mRNA 221 enrichment), and paired-end sequencing on the BGIseq500 platform were performed as previously described [35]. Raw data were processed with SOAPnuke (v1.6.5) [36] to remove adapter sequences, reads containing >1% unknown bases, and low-quality reads (>40% 224 bases with Phred score ≤ 15).

### 2.11. Statistical Analysis

All analyses were performed using GraphPad Prism 8.0.2 (GraphPad Software, San Diego, CA, USA) and R [37]. All data were shown as the mean ± standard deviation. Statistical differences among multiple groups were analyzed using one-way analysis of variance (ANOVA) or unpaired Student’s *t*-test. *p* < 0.05 was considered to indicate statistical significance.

### 2.12. RNA-Seq Data Analysis

RNA-seq data analysis was performed as follows: clean reads were aligned to the reference genome and quantified using Rsubread [38] and featureCounts. Differential expression between HFD + TRF and HFD groups was identified with DESeq2 [39] (adjusted *p* ≤ 235 0.05). The most significantly upregulated and downregulated pathways refer to those 236 with the highest positive and negative Normalized Enrichment Score (NES), respectively, from the Gene Set Enrichment Analysis (GSEA) comparing the HFD + TRF group to the HFD group. The JTK_CYCLE algorithm [40] (period = 24 h, delta = 6 h, “independent” method) was used to identify the periodic characteristics of each group, as described previously [35]. Transcripts meeting the significance threshold (BH.Q < 0.05) were deemed rhythmic. Changes in rhythm parameters (mesor, amplitude, acrophase) between the HFD + TRF and HFD groups were evaluated with CircaCompare [41], where a significant difference (*p* < 0.05) in any parameter indicated rhythm alteration. Enrichment analyses were performed using GSEA (with NES ranking pathways) and GO term analysis via clusterProfiler [42].

## 3. Results

### 3.1. Time-Restricted Feeding Significantly Restores the Circadian Rhythm of Energy Metabolism in High-Fat Diet-Fed Mice

**Figure 1:**
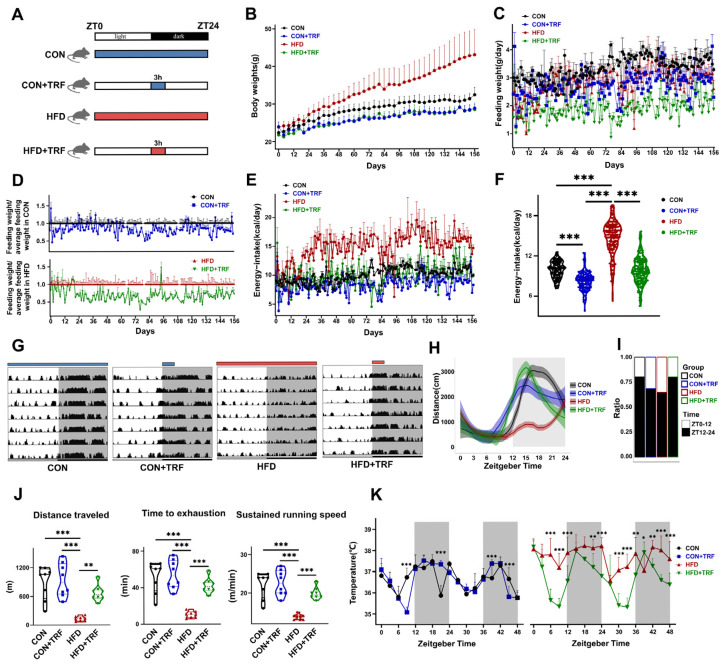
Time-restricted feeding significantly restores the circadian rhythm of energy metabolism in high-fat diet-fed mice. (**A**): Feeding pattern schematics of the four experimental mouse groups (CON: control group with normal diet, represented in black; CON + TRF: control group with time-restricted feeding, represented in blue; HFD: high-fat diet group, represented in red; HFD + TRF: high-fat diet group with time-restricted feeding, represented in green). (**B**): Line graph of body weight changes in mice (*n = 15*). (**C**): Line graph of daily food intake (by weight) in mice (*n = 3*). (**D**): Line graph of the daily average food intake ratio of TRF groups to their respective ad libitum-fed groups (CON or HFD) (*n = 3*). (**E**): Line graph of daily energy intake in mice (*n = 3*). (**F**): Violin plot showing the distribution of daily energy intake variation in mice (One-way ANOVA was used for comparisons among groups). (**G**): Bar chart of daily activity levels in mice, represented by the distance traveled per 3 min bin (shaded area indicates the dark phase; *n = 7 days*). (**H**): 24 h activity profile of mice (shaded area indicates the dark phase; *n = 7 days*). (**I**): Bar chart of the proportion of daily movement during the dark phase (black) and light phase in mice (*n = 7 days*). (**J**): Violin plot of running distance, time to exhaustion, and running speed at fatigue in mice (unpaired Student’s *t*-test was used for statistical analysis, *n = 8*). (**K**): Line graph of core body temperature fluctuations in mice over a 48 h period, measured at 3 h intervals (unpaired Student’s *t*-test was used for statistical analysis, *n = 3*). Data are presented as mean ± SD. * *p* < 0.05, ** *p* < 0.01, *** *p* < 0.001, see File S1 for details.

Sixty 8-week-old male C57BL/6 mice were randomly assigned into four groups: ad libitum standard chow-fed (CON), time-restricted feeding with standard chow (CON + TRF), ad libitum high-fat diet-fed (HFD), and time-restricted feeding with high-fat diet (HFD + TRF). CON and HFD groups had unrestricted access to food, while CON + TRF and HFD + TRF groups were fed exclusively during a 3 h window from ZT12 (19:00) to ZT15 (22:00) (Figure 1A). All groups were maintained on their respective diets for 22 weeks. HFD mice exhibited continuous weight gain, showing a significant difference from CON mice starting at week 4 (*p* < 0.05), whereas CON + TRF and HFD + TRF mice maintained body weights at ~90% of CON mice (Figure 1B). This indicates that a 3 h feeding window effectively reduces body weight in high-fat diet-fed mice to levels comparable to standard chow-fed mice, independent of dietary fat content. Daily food intake analysis showed that CON + TRF mice consumed ~16% less food than CON mice, while HFD + TRF mice consumed ~31% less than HFD mice (Figure 1C). Caloric intake analysis revealed that CON + TRF mice consumed ~83% of the calories of CON mice, and HFD mice consumed ~147% of CON calories. Notably, the HFD + TRF group exhibited caloric intake comparable to the CON group (Figure 1E,F). These findings demonstrate that a 3 h feeding window significantly reduces body weight in both standard and high-fat diet-fed mice. Additionally, the similar caloric intake of HFD + TRF and CON mice suggests that time-restricted feeding enhances energy expenditure in high-fat diet-fed mice. Energy expenditure primarily includes basal metabolic maintenance, physical activity, growth and repair, and the thermic effect of food. We monitored the locomotor activity of mice over a one-week period. As nocturnal animals, mice exhibit higher activity during the dark phase and rest during the light phase. Compared to CON mice, HFD-fed mice showed disrupted diurnal rhythms, with a loss of night-time activity and day-time rest patterns, along with a significant reduction in total daily activity (Figure 1G,H), particularly in night-time activity (Figure 1H). A 3 h TRF regimen restored the disrupted circadian rhythm induced by HFD and significantly increased night-time activity, especially during ZT12-ZT21 (Figure 1I). In summary, 3 h TRF effectively prevented circadian rhythm disruption caused by prolonged HFD feeding and significantly enhanced daily activity, particularly during the night. During the treadmill fatigue test, it was found that a high-fat diet severely impaired the exercise capacity. Compared with the control group mice, the time required for the mice in the high-fat diet group to reach the limit exercise state was reduced by approximately 80% (high-fat diet group: 10 min; control group: 52 min; *p* < 0.001). It is notable that the 3 h intermittent fasting protocol significantly improved this defect, restoring the running endurance to a level similar to that of the control group mice (high-fat diet + intermittent fasting group: 42 min; compared with the high-fat diet group, *p* < 0.001; compared with the control group, *p* > 0.05). Similarly, through intermittent fasting intervention, both the total running distance and the maximum speed also returned to normal (Figure 1J). To further explore the energy regulation mechanism, we monitored the 48 h body temperature rhythm of each group of mice. The mice in the control group (CON group) exhibited a body temperature rhythm with lower levels during the day and higher levels at night. While the mice fed with a high-fat diet (HFD) maintained their body temperature at a relatively high level of 38 °C, with a reduced amplitude of daily temperature fluctuations. Notably, the 3 h intermittent fasting (TRF) protocol effectively restored the body temperature rhythm and average body temperature of the HFD-fed mice to levels close to those of normal mice (Figure 1K).

### 3.2. TRF Significantly Improves Metabolic Dysregulation Induced by Long-Term High-Fat Diet

**Figure 2:**
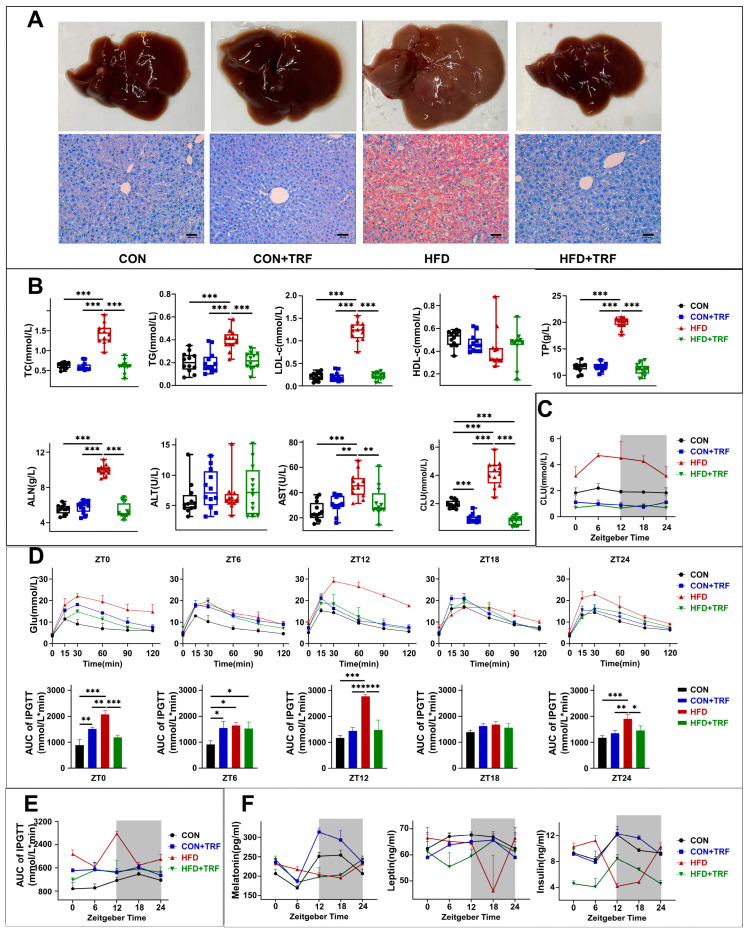
TRF significantly improves metabolic dysregulation induced by long-term high-fat diet. (**A**): Representative photographs of mouse livers and corresponding liver sections stained with Oil Red O. (**B**): Bar chart of serum biochemical parameters in mice (One-way ANOVA was used for comparisons among groups, *n = 12*). (**C**): Line graph of diurnal glucose levels in mouse serum (shaded area indicates the dark phase; *n = 3*). (**D**): Up: Line graph of glucose tolerance tests in mice. Down: Bar chart of the corresponding area under the curve (AUC) (One-way ANOVA was used for comparisons among groups, *n = 3*). (**E**): Line graph of the AUC of glucose tolerance in mice over a 24 h period (shaded area indicates the dark phase; *n = 3*). (**F**): Line graphs of diurnal variations in serum melatonin, leptin, and insulin levels in mice (shaded area indicates the dark phase; *n = 3*). Data are presented as mean ± SD. * *p* < 0.05, ** *p* < 0.01, *** *p* < 0.001 see File S2 for details.

In our subsequent experiments, we monitored the metabolic rhythms of mice. Long-term high-fat diet (HFD) significantly increased the liver weights of mice and induced hepatic steatosis, a result that was confirmed by Oil Red O staining. In contrast, 3 h time-restricted feeding (TRF) significantly reversed both the HFD-induced increase in liver weight and lipid accumulation in the liver (Figure 2A and Figure S1A,B). Additionally, 3 h time-restricted feeding (TRF) reduced the serum levels of total cholesterol (TC), triglycerides (TG), and low-density lipoprotein cholesterol (LDL-c) in mice on a high-fat diet (HFD) to normal levels (Figure 2B). It also markedly lowered the aspartate aminotransferase (AST) levels, a key marker of liver function, back to baseline. Interestingly, regardless of the diet type (standard or high-fat), 3 h TRF significantly decreased the serum glucose content in mice (Figure 2B).

Next, we assessed glucose tolerance. Continuous 24 h blood glucose monitoring revealed that the high-fat diet (HFD) group exhibited persistently elevated random blood glucose levels. In contrast, 3 h time-restricted feeding (TRF) effectively reduced random blood glucose levels in both mice that were fed a normal diet and those on an HFD (Figure 2C). When compared to the normal diet group, HFD-fed mice showed significantly higher blood glucose levels and a delayed clearance rate following glucose injection at Zeitgeber Time 0 (ZT0), ZT12, and ZT24. This resulted in a markedly increased area under the curve (AUC) for glucose tolerance tests (Figure 2D,E), indicating that long-term consumption of an HFD significantly impaired glucose tolerance. Interestingly, HFD-fed mice subjected to 3 h TRF displayed normalized glucose tolerance at all three tested time points (ZT0, ZT12, and ZT24) (Figure 2D,E). This normalization suggests that 3 h TRF can counteract the detrimental effects of an HFD on glucose tolerance. Therefore, our findings indicate that 3 h TRF not only improves glucose tolerance but also helps maintain normal blood glucose levels in mice fed an HFD, highlighting its potential as a dietary intervention for managing glucose homeostasis.

We examined the secretion of metabolism-related hormones. In the CON group, melatonin secretion was low during the light phase and elevated during the dark phase, following a typical circadian rhythm. However, HFD-fed mice lost this rhythmicity, and 3 h TRF did not significantly restore the melatonin rhythm in HFD mice (Figure 2F). In comparison to the normal diet group, the HFD group showed significantly reduced leptin levels at ZT18, while leptin levels were elevated at ZT0/24. Time-restricted feeding improved the leptin secretion rhythm. Regarding insulin secretion, TRF significantly corrected the circadian rhythm of insulin, although secretion levels at all time points remained lower than those in the normal diet group.

### 3.3. TRF Modulates HFD Induced Hepatic Gene Expression Influencing Key Metabolic Pathways

**Figure 3:**
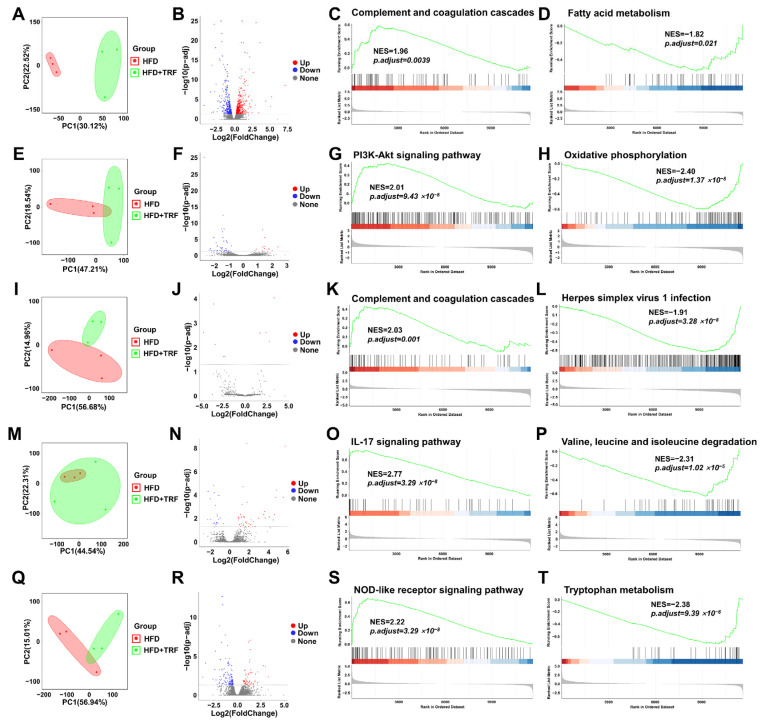
TRF modulates HFD induced hepatic gene expression influencing key metabolic pathways. (**A**,**E**,**I**,**M**,**Q**): Principal component analysis (PCA) of RNA-seq data from three samples per group at ZT0, ZT6, ZT12, ZT18, and ZT24, respectively, with the HFD group depicted in red and the HFD+TRF group depicted in green. (**B**,**F**,**J**,**N**,**R**): Volcano plots of differentially expressed genes (DEGs) at ZT0, ZT6, ZT12, ZT18, and ZT24, respectively. (**C**,**G**,**K**,**O**,**S**): Gene Set Enrichment Analysis (GSEA) plots of the most significantly upregulated pathways at ZT0, ZT6, ZT12, ZT18, and ZT24, respectively. (**D**,**H**,**L**,**P**,**T**): Gene Set Enrichment Analysis (GSEA) plots of the most significantly downregulated pathways at ZT0, ZT6, ZT12, ZT18, and ZT24, respectively. See File S3 for details.

To further investigate the effect of 3 h TRF on hepatic gene expression in HFD mice, we performed mRNA transcriptome sequencing on the livers of both HFD and HFD-TRF groups at ZT0, ZT6, ZT12, ZT18, and ZT24. Principal component analysis (PCA) of the three samples from each group at each time point revealed clear separation between the groups (Figure 3A,E,I,M,Q). Differentially expressed genes (DEGs) were identified using DESeq2 (*p*-adj < 0.05). At ZT0, TRF treatment significantly upregulated 400 genes and downregulated 355 genes (Figure 3B); at ZT6, 13 genes were upregulated and 41 downregulated (Figure 3F); at ZT12, 4 genes were upregulated and 5 downregulated (Figure 3J); at ZT18, 39 genes were upregulated and 19 downregulated (Figure 3N); and at ZT24, 56 genes were upregulated and 122 downregulated (Figure 3R). Gene Set Enrichment Analysis (GSEA) was used to detect significant pathway changes at each time point. The analysis showed that at ZT0, complement and coagulation cascades were significantly enriched and upregulated in the HFD-TRF group (Figure 3C), while fatty acid metabolism pathways were significantly downregulated (Figure 3D). At ZT6, PI3K-Akt signaling pathways were significantly enriched and upregulated in the HFD-TRF group (Figure 3G), while oxidative phosphorylation pathways were significantly downregulated (Figure 3H). At ZT12, complement and coagulation cascades were again enriched and upregulated in the HFD-TRF group (Figure 3K), while herpes simplex virus infection pathways were significantly downregulated (Figure 3L). At ZT18, IL-17 signaling pathways were significantly enriched and upregulated in the HFD-TRF group (Figure 3O), while valine, leucine, and isoleucine degradation pathways were significantly downregulated (Figure 3P). At ZT24, NOD-like receptor signaling pathways were significantly enriched and upregulated in the HFD-TRF group (Figure 3S), while tryptophan metabolism pathways were significantly downregulated (Figure 3T).

### 3.4. TRF Alters HFD-Induced Hepatic Rhythmic Gene Expression Modulating Key Metabolic Pathways

**Figure 4:**
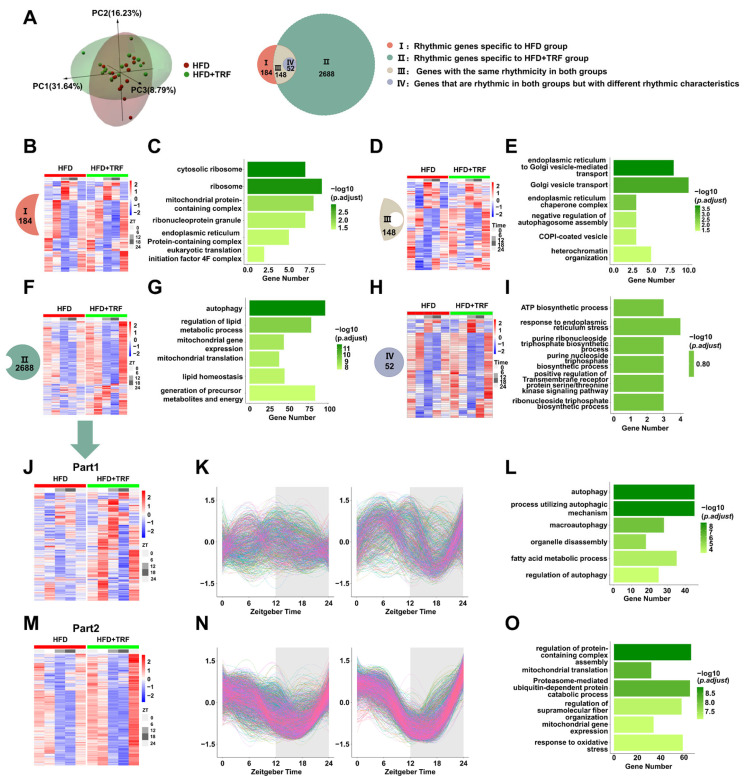
TRF alters HFD-induced hepatic rhythmic gene expression modulating key metabolic pathways. (**A**): The 3D PCA diagrams of the two groups of samples (**left**) and the Venn diagram of the rhythmic gene set (**right**). (**B**,**D**,**F**,**H**): Heatmaps of diurnal expression for the four distinct clusters of rhythmic genes delineated in Figure 3A, respectively. (**C**,**E**,**G**,**I**): Significantly enriched Gene Ontology (GO) terms for the rhythmic genes from the corresponding clusters in Figure 3A. (**J**,**M**): Heatmaps of expression patterns for the two distinct subclusters derived from the gene cluster in Figure 3F. (**K**,**N**): Curve fitting of the diurnal expression patterns for genes in subclusters (**J**,**M**), respectively (each line represents an individual gene; left panel: HFD group, right panel: HFD + TRF group; shaded area indicates the dark phase). (**L**,**O**): Significantly enriched Gene Ontology (GO) terms for genes in subclusters (**J**,**M**), respectively.

PCA of liver RNA-seq data from the HFD and HFD + TRF groups revealed a clear separation between the two groups (Figure 4A), indicating a profound and systemic reprogramming of the hepatic transcriptome by the 3 h TRF intervention. We then compared the rhythmic transcriptomes of liver tissues from HFD and HFD-TRF mice using JTK-cycle rhythmicity analysis, with genes having an adjusted *p*-value (*p*-adj) < 0.05 considered rhythmic. For shared rhythmic genes between the two groups, we used the CircaCompare method to compare median, amplitude, and phase differences. Genes with at least one *p*-value < 0.05 for median, amplitude, or phase were classified as having rhythmic changes, while genes with *p*-values ≥ 0.05 in all three parameters were considered unchanged. Analysis revealed that 3 h TRF dramatically increased the number of rhythmic genes in HFD-fed mice. The HFD group had 184 unique rhythmic genes (Group I), while the HFD-TRF group had 2688 unique rhythmic genes (Group II), representing a ~14.6-fold induction and underscoring the potent circadian-reinforcing effect of extreme TRF. There were 148 shared unchanged rhythmic genes (Group III) and 52 shared changed rhythmic genes (Group IV) (Figure 4A,B,D,F,H). Notably, genes in Group I were significantly enriched in pathways related to ribosome biogenesis and basic cellular “housekeeping” functions (e.g., mitochondrial and ER protein complexes) (Figure 4C), suggesting that under metabolic stress, a minimal set of core cellular processes retains circadian regulation. Group III genes showed similar rhythmic oscillations in both groups (Figure 4D), and were enriched in pathways related to ER-to-Golgi vesicle-mediated transport, Golgi vesicle transport, and ER chaperone complexes (Figure 4E). In contrast, the vast majority of genes that gained robust rhythmicity under TRF (Group II) were enriched in central metabolic pathways such as autophagy, lipid metabolism regulation, and mitochondrial gene expression (Figure 4G). This stark dichotomy strongly indicates that TRF specifically restores circadian timing to metabolic pathways critical for energy homeostasis, which are otherwise dysregulated by HFD. The small subset of genes whose rhythm parameters were altered (Group IV) were linked to ATP biosynthesis and ER stress response (Figure 4I), hinting at TRF’s role in modulating energetic and proteostatic rhythms. We aimed to investigate how time restricted feeding induces rhythmic gene expression under metabolic stress conditions. Using the heatmap clustering with the Ward. D method applied to gene expression patterns, we identified that 2688 genes in Group II could be further subdivided into two distinct subgroups, designated II-1 and II-2 (Figure 4F). Subgroup II-1 (893 genes) transitioned from complete arrhythmia under HFD to acquiring de novo circadian rhythmicity after TRF (Figure 4J,K). These genes were selectively enriched in autophagy and fatty acid metabolism (Figure 4L), implying that TRF can act as a strong zeitgeber to “re-clock” and synchronize these key catabolic pathways. Conversely, subgroup II-2 (1795 genes) showed preexisting, low-amplitude oscillations under HFD that were significantly amplified by TRF (Figure 4M,N). These were linked to protein complex assembly and mitochondrial translation (Figure 4O), suggesting that TRF enhances the amplitude of nascent or dampened metabolic rhythms, thereby reinforcing temporal compartmentalization. Collectively, these data reveal that TRF employs at least two distinct transcriptional strategies—de novo induction and amplitude amplification—to achieve comprehensive intra-pathway synchronization, which likely underlies its efficacy in restoring metabolic efficiency.

### 3.5. TRF Restores Synchronized Circadian Rhythms in Key Liver Metabolic Pathways Disrupted by a High-Fat Diet

**Figure 5:**
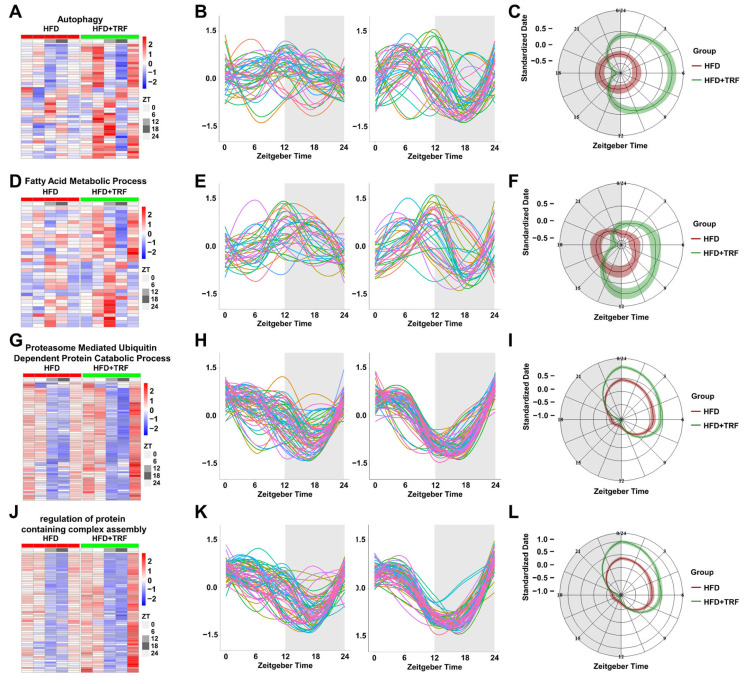
TRF restores synchronized circadian rhythms in key liver metabolic pathways disrupted by a high-fat diet. (**A**): Heatmap of diurnal expression for genes from Figure 4L significantly enriched in the autophagy pathway. (**B**): Curve fitting of the diurnal expression patterns for genes in panel (**A**) ((**left**): HFD group; (**right**): HFD + TRF group; each line represents an individual gene; shaded area indicates the dark phase). (**C**): Radar chart of diurnal expression for genes in panel (**A**). (**D**): Heatmap of diurnal expression for genes from Figure 4L significantly enriched in the Fatty Acid Metabolic Process pathway. (**E**): Curve fitting of the diurnal expression patterns for genes in panel (**D**) ((**left**): HFD group; (**right**): HFD + TRF group; each line represents an individual gene; shaded area indicates the dark phase). (**F**): Radar chart of diurnal expression for genes in panel (**D**). (**G**): Heatmap of diurnal expression for genes from Figure 4O significantly enriched in the Proteasome-mediated Ubiquitin-dependent Protein Catabolic Process pathway. (**H**): Curve fitting of the diurnal expression patterns for genes in panel (**G**) (**left**): HFD group; (**right**): HFD + TRF group; each line represents an individual gene; shaded area indicates the dark phase). (**I**): Radar chart of diurnal expression for genes in panel (**G**). (**J**): Heatmap of diurnal expression for genes from Figure 4O significantly enriched in the Regulation of Protein-containing Complex Assembly pathway. (**K**): Curve fitting of the diurnal expression patterns for genes in panel (**J**) ((**left**): HFD group; (**right**): HFD + TRF group; each line represents an individual gene; shaded area indicates the dark phase). (**L**): Radar chart of diurnal expression for genes in panel (**J**).

To further investigate the underlying mechanisms, we performed an in-depth analysis of the key pathways enriched among TRF-induced rhythmic genes. Analysis of 45 genes significantly enriched in the autophagy pathway revealed that, compared to the arrhythmic expression patterns observed in the HFD group, most genes in the TRF group exhibited a peak at ZT6 and a trough at ZT18 (Figure 5A–C). Similarly, analysis of 35 genes enriched in the fatty acid metabolic process pathway showed that, unlike the arrhythmic expression patterns observed in the HFD group, most genes in the TRF group peaked at ZT12 and reached a trough at ZT18 (Figure 5D–F). Analysis of genes enriched in the proteasome-mediated ubiquitin-dependent protein catabolic process and regulation of protein containing complex assembly pathways revealed that, under long term 3 h of TRF treatment, expression amplitudes were increased and phase synchrony was enhanced, with peaks around ZT3 and troughs near ZT15 (Figure 5G–L). These findings suggest that time-restricted feeding promotes circadian synchronization of genes within key metabolic and proteostatic pathways. The increased amplitude and precise temporal alignment likely enable cells to perform specific functions more efficiently within defined time windows, minimizing unnecessary energy expenditure and enhancing overall metabolic economy under conditions of stress.

### 3.6. TRF Synchronizes the Expression of a Large Number of Metabolic Genes

**Figure 6:**
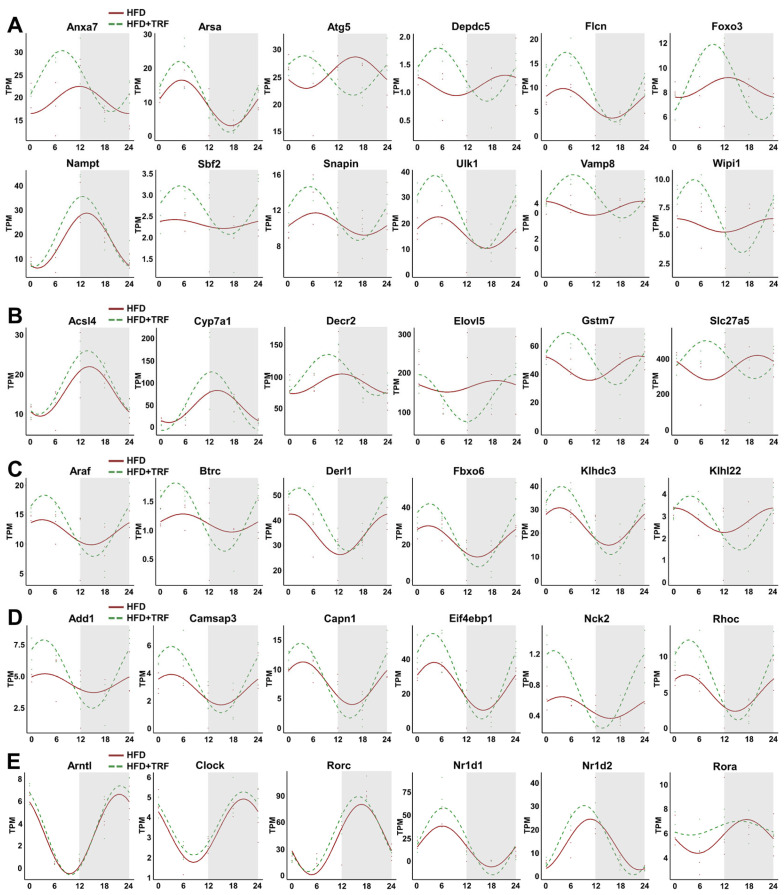
TRF restores synchronized circadian rhythms in key liver metabolic pathways disrupted by a high-fat diet. (**A**): Line graph of the fitted expression curves for genes in the autophagy pathway (shaded area indicates the dark phase). (**B**): Line graph of the fitted expression curves for genes in the Fatty Acid Metabolic Process pathway (shaded area indicates the dark phase). (**C**): Line graph of the fitted expression curves for genes in the Proteasome-mediated Ubiquitin-dependent Protein Catabolic Process pathway (shaded area indicates the dark phase). (**D**): Line graph of the fitted expression curves for genes in the Regulation of Protein-containing Complex Assembly pathway (shaded area indicates the dark phase). (**E**): Line graph of the fitted expression curves for core circadian genes (shaded area indicates the dark phase).

To further investigate these enriched pathways, we generated expression profiles of pathway-specific genes using the CircaCompare algorithm to assess circadian parameters such as phase, amplitude, and MESOR. Consistent with Figure 5A–C, genes in the autophagy pathway predominantly peaked at ZT6 and displayed pronounced rhythmic oscillations, including *Anxa7*, *Atg5*, *Foxo3*, and *Ulk1* (Figure 6A). However, a subset of autophagy-related genes, such as *Nampt*, showed peak expression at ZT12, indicating some degree of phase heterogeneity within the pathway. In the fatty acid metabolism pathway, most genes reached peak expression at ZT12 with evident rhythmic oscillations, including *Acsl4*, *Cyp7a1*, and *Decr2* (Figure 6B). A few genes, such as *Elovl5*, showed troughs at ZT12, whereas others, including *Gstm7* and *Slc27a5*, showed peak expression near ZT6 (Figure 6B). Genes involved in the proteasome-mediated ubiquitin-dependent protein catabolic process generally reached their expression peak at ZT3 with significant rhythmic oscillations; examples include Araf, Btrc, and Derl1 (Figure 6C). Similarly, genes in the regulation of protein-containing complex assembly pathway also peaked at ZT3 and exhibited robust rhythmic oscillations, such as Add1, Camsap3, and Capn1 (Figure 6D). Given these observations, we speculate that the 3 h TRF regimen induces a core clock component to participate in regulating the coordinated oscillation of genes within the significantly enriched pathways, thereby enabling the liver to perform metabolic functions in an efficient, coordinated manner. Consequently, we analyzed the diurnal expression of core clock genes. Among them, genes such as Arntl, Clock, and Rorc showed no significant expression differences, whereas Rora, Nr1d2, and Nr1d1 exhibited more pronounced alterations (Figure 6E), suggesting that these core circadian genes may play important roles in the TRF-mediated improvement of liver function.

In summary, long-term 3 h TRF intervention in high-fat-fed mice not only markedly increased the number of rhythmically expressed hepatic genes but also, for the first time, induced a higher-order reorganization of the circadian transcriptome characterized by intra-pathway synchronization. This synchronization led to coherent peak expression of functionally related genes within defined temporal windows: autophagy-related genes predominantly at ZT6, fatty acid metabolic genes at ZT12, and proteostasis-related genes around ZT3. Notably, this temporal restructuring was accompanied by altered expression of specific core clock genes (e.g., Rora, Nr1d1), suggesting a potential molecular link between the feeding regimen and the observed transcriptional reprogramming. Collectively, these findings demonstrate that stringent TRF promotes a more robust and temporally precise circadian regulation of hepatic metabolism, which likely underlies the restoration of metabolic homeostasis disrupted by a high-fat diet.

## 4. Discussion

In this study, we demonstrate that a 3 h time-restricted feeding (TRF) regimen significantly restores disrupted circadian rhythms and ameliorates metabolic dysfunctions induced by a long-term high-fat diet (HFD) in mice. Specifically, TRF improves rhythmic locomotor activity, energy expenditure, glucose tolerance, and lipid homeostasis while reestablishing circadian body temperature rhythms. Transcriptomic profiling reveals that TRF reprograms hepatic gene expression, not only increasing the number of rhythmically expressed genes but, for the first time, demonstrating that TRF induces intra-pathway synchronization of rhythmic gene expression. This synchronization, observed within key metabolic and proteostatic pathways such as autophagy, fatty acid metabolism, and protein degradation, suggests a novel mechanism through which TRF enhances cellular efficiency under metabolic stress.

Our findings align with and extend previous studies that highlight the metabolic benefits of TRF under obesogenic conditions. Hatori et al. [43] and Chaix et al. [20] reported that TRF improves metabolic parameters such as insulin sensitivity and hepatic lipid accumulation without reducing caloric intake. More recently, Guan et al. [44] and Qu et al. [45] demonstrated that TRF reprograms the hepatic circadian transcriptome and enhances the rhythmic expression of genes involved in lipid metabolism and inflammation. Consistent with these observations, our study confirms that TRF increases the number of rhythmically expressed genes in the liver and further reveals that TRF promotes temporal synchronization within key metabolic pathways, including autophagy, fatty acid metabolism, and proteostasis. Importantly, our study is the first to demonstrate that TRF not only induces circadian rhythmicity in previously arrhythmic genes under HFD conditions but also drives synchronization of gene expression within individual metabolic pathways. This intra-pathway synchronization, characterized by genes peaking within narrow temporal windows, has not been previously reported and represents a novel layer of circadian coordination. This coordinated rhythmicity likely enhances cellular efficiency by aligning metabolic demands with optimal timing. This concept aligns with the emerging view that TRF acts as a potent zeitgeber to reprogram circadian genomes and metabolic programs across tissues [46]. Moreover, most previous studies have applied TRF windows ranging from 6 to 12 h [11,12,15,16,19]. In contrast, our study uniquely employed a stringent 3 h TRF regimen, which has not been previously reported. Despite the shorter feeding window, we observed significant improvements in metabolic parameters and robust reprogramming of circadian gene expression. These findings suggest that even narrow feeding windows can produce substantial physiological benefits. However, the 3 h time-restricted feeding pattern is overly stringent. Given human dietary habits and adherence, it is difficult to achieve clinical intervention translation. A substantial amount of research is still required to identify the optimal balance point between efficacy and feasibility for time-restricted eating windows.

The synchronization of circadian gene expression within metabolic pathways suggests that TRF optimizes the temporal allocation of cellular resources. By concentrating gene expression peaks within specific Zeitgeber times, TRF may reduce transcriptional noise and energy waste, promoting metabolic efficiency. This principle of “temporal compartmentalization” to optimize resource use is supported by recent findings that circadian rhythms function to minimize the bioenergetic cost of maintaining protein homeostasis through coordinated, time-of-day-specific cycles of protein synthesis and degradation [47]. For instance, in the autophagy pathway, which is essential for cellular degradation and recycling processes, TRF induced a striking alignment of gene expression peaks at ZT6. This is particularly relevant as autophagy is recognized as a tightly circadian-controlled process [23]. Genes such as Anxa7, Atg5, Foxo3, and Ulk1 displayed robust rhythmicity and temporal coherence under TRF conditions, in contrast to the arrhythmic patterns observed under HFD. This pathway-level synchronization likely enables hepatocytes to conduct autophagic functions efficiently during a defined window of the circadian cycle. Similarly, in the fatty acid metabolism and protein degradation pathways, we observed comparable temporal alignment of gene expression. These synchronized rhythms within each pathway may be crucial for enabling hepatocytes to coordinate energy production, substrate utilization, and proteostatic maintenance during metabolic stress. The emergence of synchronized gene activity within functionally related pathways likely reflects a strategic adaptation to enhance cellular efficiency and minimize energetic waste. This intra-organ coordination may extend beyond the liver, as TRF has been shown to resynchronize rhythmic oscillations of the gut microbiota, which in turn drives metabolic improvements in a time-of-day-specific manner, highlighting the systemic nature of temporal reorganization [48,49]. Such optimized timing may represent an adaptive mechanism by which organisms conserve energy under metabolic stress, and it highlights synchronization as a critical dimension of TRF’s therapeutic effect—distinct from mere restoration of rhythmicity. An important open question raised by our findings is whether this intra-pathway synchronization is orchestrated by specific circadian transcription factors that act as local regulators of rhythmic coherence. This is plausible given that fine-tuning of clock protein (e.g., DBP) levels through regulated degradation is a known mechanism for shaping circadian transcriptome dynamics and downstream metabolic outputs [50]. Identifying such regulators will be a key focus of future work aimed at uncovering the molecular basis of TRF-induced temporal reorganization.

These findings have important physiological and translational implications. The robustness of these conclusions is underpinned by the multidisciplinary design of our study. Our integrated approach, encompassing behavioral phenotyping (locomotor activity, treadmill tests), continuous physiological monitoring (core body temperature rhythms), and high-resolution temporal transcriptomics (RNA-seq across five circadian time points), provided convergent and mutually reinforcing evidence. This convergence across different levels of biological organization—from whole-animal behavior to systemic physiology and to hepatic gene networks—strengthens the validity of our central finding that extreme TRF acts as a powerful synchronizer, restoring temporal coherence not just within the liver transcriptome but across multiple circadian output pathways.

However, several limitations of this study should be acknowledged to guide future research. First, while our transcriptomic analysis focused on delineating the therapeutic mechanism of TRF in reversing HFD-induced dysfunction, the absence of RNA-seq data from the CON and CON + TRF groups limits a direct comparison of how TRF reshapes the hepatic transcriptome in healthy versus pathological states. Second, although the HFD-fed mice exhibited hallmark metabolic and histological signs of NAFLD (e.g., steatosis via Oil Red O staining), a more comprehensive histological evaluation, including H&E staining for hepatocyte ballooning and inflammation, would further strengthen the phenotypic characterization. Third, our sampling at 6 h intervals, while standard for capturing core circadian oscillations, may have missed finer transcriptional dynamics. Future studies employing denser time-series (e.g., 3 h intervals) could reveal more precise phase relationships within the synchronized pathways we identified.

Beyond these methodological considerations, circadian misalignment is a hallmark of metabolic syndrome and non-alcoholic fatty liver disease (NAFLD) [51,52]. By reestablishing synchronized circadian gene expression, TRF could offer a non-pharmacological intervention to mitigate circadian dysfunction-associated diseases [43]. Notably, TRF restored glucose tolerance and lipid profiles to levels comparable to control mice, highlighting its therapeutic potential in metabolic disorders [53]. Additionally, the enhancement of rhythmic gene expression under TRF suggests that circadian-based feeding regimens may improve the efficacy of chronotherapy by aligning drug administration with optimal gene expression windows. This aligns with the broader principle that timing drug administration to coincide with optimal gene expression windows can enhance treatment outcomes [54,55]. Although hepatic transcriptomic changes were comprehensively characterized, the role of other metabolic organs (e.g., adipose tissue, skeletal muscle) in mediating the systemic effects of TRF remains unclear. Future studies incorporating multi-organ temporal profiling and single-cell resolution may further elucidate the systemic circadian reprogramming induced by TRF. Finally, the translatability of our findings to human populations requires investigation, including whether shorter or less stringent feeding windows produce comparable benefits under real-world conditions.

In summary, this study provides compelling evidence that TRF serves as an effective circadian reprogrammer that mitigates HFD-induced metabolic dysfunction by restoring rhythmic gene expression in liver metabolic pathways. Notably, we provide the first evidence that TRF induces intra-pathway synchronization of gene expression in autophagy, fatty acid metabolism, and proteostasis—an adaptive temporal alignment that may enhance hepatic efficiency under metabolic stress. These findings lay the groundwork for future translational research into TRF-based interventions for metabolic diseases.

## Data Availability

The Total RNA-seq data is stored in the China National Center for Bioinformation database (https://www.cncb.ac.cn/) with the project number ID PRJCA043041. Any additional information required to reanalyze the data reported will be shared by the lead contact upon request.

## Supplementary Materials

The following supporting information can be downloaded at: https://www.mdpi.com/article/10.3390/cells15020193/s1, File S1. Data and statistical analysis related to Figure 1; File S2. Data and statistical analysis related to Figure 2; File S3. Data and statistical analysis related to Figure 3; File S4. Data and statistical analysis related to Figure S1; Figure S1. A: Violin plot of liver weights in four groups of mice, n = 15. B: Box plot of the quantified liver Oil Red O staining area as a proportion of the section area in four groups of mice, n = 9. One-way ANOVA was employed for statistical analysis among the groups. Data are presented as mean ± SD. * *p* < 0.05, ** *p* < 0.01, *** *p* < 0.001.

## References

[1] Younossi Z.M., Loomba R., Rinella M.E., Bugianesi E., Marchesini G., Neuschwander-Tetri B.A., Serfaty L., Negro F., Caldwell S.H., Ratziu V. (2018). Current and future therapeutic regimens for nonalcoholic fatty liver disease and nonalcoholic steatohepatitis. Hepatology.

[2] Friedman S.L., Neuschwander-Tetri B.A., Rinella M., Sanyal A.J. (2018). Mechanisms of NAFLD development and therapeutic strategies. Nat. Med..

[3] Rinella M.E. (2015). Nonalcoholic fatty liver disease: A systematic review. JAMA.

[4] Jacobi D., Liu S., Burkewitz K., Kory N., Knudsen N.H., Alexander R.K., Unluturk U., Li X., Kong X., Hyde A.L. (2015). Hepatic Bmal1 Regulates Rhythmic Mitochondrial Dynamics and Promotes Metabolic Fitness. Cell Metab..

[5] Daniels L.J., Kay D., Marjot T., Hodson L., Ray D.W. (2023). Circadian regulation of liver metabolism: Experimental approaches in human, rodent, and cellular models. Am. J. Physiol. Cell Physiol..

[6] Sato T., Sassone-Corsi P. (2022). Nutrition, metabolism, and epigenetics: Pathways of circadian reprogramming. EMBO Rep..

[7] Martinez-Sanchez N., Ray D. (2024). Rhythmic liver drives feeding behavior. Science.

[8] Perez-Diaz-Del-Campo N., Castelnuovo G., Caviglia G.P., Armandi A., Rosso C., Bugianesi E. (2022). Role of Circadian Clock on the Pathogenesis and Lifestyle Management in Non-Alcoholic Fatty Liver Disease. Nutrients.

[9] Liu M., Zhang Z., Chen Y., Feng T., Zhou Q., Tian X. (2023). Circadian clock and lipid metabolism disorders: A potential therapeutic strategy for cancer. Front. Endocrinol..

[10] Luo Y., Meng X., Cui L., Wang S. (2024). Circadian Regulation of Lipid Metabolism during Pregnancy. Int. J. Mol. Sci..

[11] Pappachan J.M. (2024). In T2DM with obesity, time-restricted eating increased weight loss and reduced HbA(1c) level at 6 mo. Ann. Intern. Med..

[12] Harris E. (2023). Time-Restricted Eating Tested for Weight Loss in Type 2 Diabetes. JAMA.

[13] Cheng W.Y., Desmet L., Depoortere I. (2023). Time-restricted eating for chronodisruption-related chronic diseases. Acta Physiol..

[14] Xie Z., Sun Y., Ye Y., Hu D., Zhang H., He Z., Zhao H., Yang H., Mao Y. (2022). Randomized controlled trial for time-restricted eating in healthy volunteers without obesity. Nat. Commun..

[15] Wilkinson M.J., Manoogian E.N.C., Zadourian A., Lo H., Fakhouri S., Shoghi A., Wang X., Fleischer J.G., Navlakha S., Panda S. (2020). Ten-Hour Time-Restricted Eating Reduces Weight, Blood Pressure, and Atherogenic Lipids in Patients with Metabolic Syndrome. Cell Metab..

[16] Sutton E.F., Beyl R., Early K.S., Cefalu W.T., Ravussin E., Peterson C.M. (2018). Early Time-Restricted Feeding Improves Insulin Sensitivity, Blood Pressure, and Oxidative Stress Even without Weight Loss in Men with Prediabetes. Cell Metab..

[17] Acosta-Rodríguez V.A., de Groot M.H.M., Rijo-Ferreira F., Green C.B., Takahashi J.S. (2017). Mice under Caloric Restriction Self-Impose a Temporal Restriction of Food Intake as Revealed by an Automated Feeder System. Cell Metab..

[18] Mitchell S.J., Bernier M., Mattison J.A., Aon M.A., Kaiser T.A., Anson R.M., Ikeno Y., Anderson R.M., Ingram D.K., de Cabo R. (2019). Daily Fasting Improves Health and Survival in Male Mice Independent of Diet Composition and Calories. Cell Metab..

[19] Chaix A., Deota S., Bhardwaj R., Lin T., Panda S. (2021). Sex- and age-dependent outcomes of 9-hour time-restricted feeding of a Western high-fat high-sucrose diet in C57BL/6J mice. Cell Rep..

[20] Chaix A., Zarrinpar A., Miu P., Panda S. (2014). Time-restricted feeding is a preventative and therapeutic intervention against diverse nutritional challenges. Cell Metab..

[21] Escalante-Covarrubias Q., Mendoza-Viveros L., Gonzalez-Suarez M., Sitten-Olea R., Velazquez-Villegas L.A., Becerril-Perez F., Pacheco-Bernal I., Carreno-Vazquez E., Mass-Sanchez P., Bustamante-Zepeda M. (2023). Time-of-day defines NAD(+) efficacy to treat diet-induced metabolic disease by synchronizing the hepatic clock in mice. Nat. Commun..

[22] Guan D., Xiong Y., Trinh T.M., Xiao Y., Hu W., Jiang C., Dierickx P., Jang C., Rabinowitz J.D., Lazar M.A. (2020). The hepatocyte clock and feeding control chronophysiology of multiple liver cell types. Science.

[23] Ulgherait M., Midoun A.M., Park S.J., Gatto J.A., Tener S.J., Siewert J., Klickstein N., Canman J.C., Ja W.W., Shirasu-Hiza M. (2021). Circadian autophagy drives iTRF-mediated longevity. Nature.

[24] Chaix A., Lin T., Le H.D., Chang M.W., Panda S. (2019). Time-Restricted Feeding Prevents Obesity and Metabolic Syndrome in Mice Lacking a Circadian Clock. Cell Metab..

[25] Hepler C., Weidemann B.J., Waldeck N.J., Marcheva B., Cedernaes J., Thorne A.K., Kobayashi Y., Nozawa R., Newman M.V., Gao P. (2022). Time-restricted feeding mitigates obesity through adipocyte thermogenesis. Science.

[26] Manella G., Sabath E., Aviram R., Dandavate V., Ezagouri S., Golik M., Adamovich Y., Asher G. (2021). The liver-clock coordinates rhythmicity of peripheral tissues in response to feeding. Nat. Metab..

[27] Bass J. (2024). Interorgan rhythmicity as a feature of healthful metabolism. Cell Metab..

[28] Woodie L.N., Alberto A.J., Krusen B.M., Melink L.C., Lazar M.A. (2024). Genetic synchronization of the brain and liver molecular clocks defend against chrono-metabolic disease. Proc. Natl. Acad. Sci. USA.

[29] Petrus P., Smith J.G., Koronowski K.B., Chen S., Sato T., Greco C.M., Mortimer T., Welz P.S., Zinna V.M., Shimaji K. (2022). The central clock suffices to drive the majority of circulatory metabolic rhythms. Sci. Adv..

[30] Schrader L.A., Ronnekleiv-Kelly S.M., Hogenesch J.B., Bradfield C.A., Malecki K.M. (2024). Circadian disruption, clock genes, and metabolic health. J. Clin. Investig..

[31] Dong Y., Lam S.M., Li Y., Li M.D., Shui G. (2025). The circadian clock at the intersection of metabolism and aging—Emerging roles of metabolites. J. Genet. Genom..

[32] Vollmers C., Gill S., DiTacchio L., Pulivarthy S.R., Le H.D., Panda S. (2009). Time of feeding and the intrinsic circadian clock drive rhythms in hepatic gene expression. Proc. Natl. Acad. Sci. USA.

[33] Deota S., Lin T., Chaix A., Williams A., Le H., Calligaro H., Ramasamy R., Huang L., Panda S. (2023). Diurnal transcriptome landscape of a multi-tissue response to time-restricted feeding in mammals. Cell Metab..

[34] Dougherty J.P., Springer D.A., Gershengorn M.C. (2016). The Treadmill Fatigue Test: A Simple, High-throughput Assay of Fatigue-like Behavior for the Mouse. J. Vis. Exp..

[35] Liu S., Zhuo K., Wang Y., Wang X., Zhao Y. (2024). Prolonged Sleep Deprivation Induces a Reprogramming of Circadian Rhythmicity with the Hepatic Metabolic Transcriptomic Profile. Biology.

[36] Chen Y., Chen Y., Shi C., Huang Z., Zhang Y., Li S., Li Y., Ye J., Yu C., Li Z. (2018). SOAPnuke: A MapReduce acceleration-supported software for integrated quality control and preprocessing of high-throughput sequencing data. Gigascience.

[37] The R Team (2014). R: A Language and Environment for Statistical Computing.

[38] Liao Y., Smyth G.K., Shi W. (2019). The R package Rsubread is easier, faster, cheaper and better for alignment and quantification of RNA sequencing reads. Nucleic Acids Res..

[39] Love M.I., Huber W., Anders S. (2014). Moderated estimation of fold change and dispersion for RNA-seq data with DESeq2. Genome Biol..

[40] Hughes M.E., Hogenesch J.B., Kornacker K. (2010). JTK_CYCLE: An efficient nonparametric algorithm for detecting rhythmic components in genome-scale data sets. J. Biol. Rhythm..

[41] Parsons R., Parsons R., Garner N., Oster H., Rawashdeh O. (2020). CircaCompare: A method to estimate and statistically support differences in mesor, amplitude and phase, between circadian rhythms. Bioinformatics.

[42] Wu T., Hu E., Xu S., Chen M., Guo P., Dai Z., Feng T., Zhou L., Tang W., Zhan L. (2021). clusterProfiler 4.0: A universal enrichment tool for interpreting omics data. Innovation.

[43] Hatori M., Vollmers C., Zarrinpar A., DiTacchio L., Bushong E.A., Gill S., Leblanc M., Chaix A., Joens M., Fitzpatrick J.A. (2012). Time-restricted feeding without reducing caloric intake prevents metabolic diseases in mice fed a high-fat diet. Cell Metab..

[44] Guan D., Lazar M.A. (2022). Circadian Regulation of Gene Expression and Metabolism in the Liver. Semin. Liver. Dis..

[45] Frazier K., Manzoor S., Carroll K., DeLeon O., Miyoshi S., Miyoshi J., St George M., Tan A., Chrisler E.A., Izumo M. (2023). Gut microbes and the liver circadian clock partition glucose and lipid metabolism. J. Clin. Invest..

[46] Xie X., Zhang M., Luo H. (2024). Regulation of metabolism by circadian rhythms: Support from time-restricted eating, intestinal microbiota & omics analysis. Life Sci..

[47] Seinkmane E., Edmondson A., Peak-Chew S.Y., Zeng A., Rzechorzek N.M., James N.R., West J., Munns J., Wong D.C., Beale A.D. (2024). Circadian regulation of macromolecular complex turnover and proteome renewal. EMBO J..

[48] Xia J., Guo W., Hu M., Jin X., Zhang S., Liu B., Qiu H., Wang K., Zhuge A., Li S. (2023). Resynchronized rhythmic oscillations of gut microbiota drive time-restricted feeding induced nonalcoholic steatohepatitis alleviation. Gut Microbes.

[49] Palomba A., Tanca A., Abbondio M., Sau R., Serra M., Marongiu F., Fraumene C., Pagnozzi D., Laconi E., Uzzau S. (2021). Time-restricted feeding induces Lactobacillus- and Akkermansia-specific functional changes in the rat fecal microbiota. NPJ Biofilms Microbiomes.

[50] Masuda S., Kurabayashi N., Nunokawa R., Otobe Y., Kozuka-Hata H., Oyama M., Shibata Y., Inoue J.I., Koebis M., Aiba A. (2024). TRAF7 determines circadian period through ubiquitination and degradation of DBP. Commun. Biol..

[51] Koike N., Umemura Y., Inokawa H., Tokuda I., Tsuchiya Y., Sasawaki Y., Umemura A., Masuzawa N., Yabumoto K., Seya T. (2024). Inter-individual variations in circadian misalignment-induced NAFLD pathophysiology in mice. iScience.

[52] Verdelho Machado M. (2024). Circadian Deregulation: Back Facing the Sun Toward Metabolic Dysfunction-Associated Steatotic Liver Disease (MASLD) Development. Nutrients.

[53] Sinturel F., Chera S., Brulhart-Meynet M.C., Montoya J.P., Stenvers D.J., Bisschop P.H., Kalsbeek A., Guessous I., Jornayvaz F.R., Philippe J. (2023). Circadian organization of lipid landscape is perturbed in type 2 diabetic patients. Cell Rep. Med..

[54] Sulli G., Manoogian E.N.C., Taub P.R., Panda S. (2018). Training the Circadian Clock, Clocking the Drugs, and Drugging the Clock to Prevent, Manage, and Treat Chronic Diseases. Trends Pharmacol. Sci..

[55] Pferdehirt L., Damato A.R., Lenz K.L., Gonzalez-Aponte M.F., Palmer D., Meng Q.J., Herzog E.D., Guilak F. (2025). A synthetic chronogenetic gene circuit for programmed circadian drug delivery. Nat. Commun..

